# Railway Surgery

**Published:** 1890-10

**Authors:** 


RAILWAY SURGERY. A Practical Work on the Special Department of Railway Surgery; for Railway Surgeons and Practitioners in the General Practice of Surgery. By C. B. STEMEN, A. M. M. D., LL. D., Professor of Surgery in the Fort Wayne College of Medicine; Surgeon to the St. Joseph Hospital, etc. With numerous illustrations. 8vo, pp. vii.—315. 1890. J. H. Chambers & Co.

In his preface the author states his belief that a treatise upon Railway Surgery was urgently demanded, an opinion to which many surgeons will take exception. In fact, there is little in this work which may not be found in the completer surgical works. Still, the book contains much practical information, evidently written by a man of experience in surgery — not literature. There are a good many little hints which every surgeon who has had any experience with cases of railroad injury will appreciate. It is from a clinical point alone, however, that the book deserves recognition. There is much "padding" with quotations, and sometimes with subjects quite unrelated to railroad surgery. The author occasionally seems somewhat rusty, as, for instance, when he makes the statement as a histological fact that in the bones "in old persons the mineral (matter) greatly preponderates," and such grammatical delinquencies as "a complete fracture is where, etc," abound. In fact, the book is full of rudely constructed sentences and mis-spelled words. It is, indeed, hard to reconcile such a specimen of English with the degrees A. M. and LL. D., which are associated with the author's name. The publishers have done their work well. The cuts are good, the type clear, and the whole dress of the work merits praise. J. P.

